# Factors Influencing Patients’ Initial Decisions Regarding Telepsychiatry Participation During the COVID-19 Pandemic: Telephone-Based Survey

**DOI:** 10.2196/25469

**Published:** 2020-12-22

**Authors:** Jennifer Severe, Ruiqi Tang, Faith Horbatch, Regina Onishchenko, Vidisha Naini, Mary Carol Blazek

**Affiliations:** 1 Department of Psychiatry University of Michigan Ann Arbor, MI United States; 2 University of Michigan Medical School Ann Arbor, MI United States

**Keywords:** telepsychiatry, COVID-19, video visit, telephone visit, telehealth, mental health, United States, decision making, virtual care

## Abstract

**Background:**

Telepsychiatry enables patients to establish or maintain psychiatric care during the COVID-19 pandemic. Little is known about the factors influencing patients’ initial decisions to participate in telepsychiatry in the midst of a public health crisis.

**Objective:**

This paper seeks to examine factors influencing patients’ initial decisions to accept or decline telepsychiatry immediately after the stay-at-home order in Michigan, their initial choice of virtual care modality (video or telephone), and their anticipated participation in telepsychiatry once clinics reopen for in-person visits.

**Methods:**

Between June and August 2020, we conducted a telephone-based survey using a questionnaire comprising 14 quantitative and two qualitative items as part of a quality improvement initiative. We targeted patients who had an in-person appointment date that fell in the first few weeks following the Michigan governor’s stay-at-home order, necessitating conversion to virtual visits or deferment of in-person care. We used descriptive statistics to report individual survey responses and assess the association between chosen visit type and patient characteristics and future participation in telepsychiatry using multivariable logistic regression.

**Results:**

A total of 244 patients whose original in-person appointments were scheduled within the first 3 weeks of the stay-at-home order in Michigan completed the telephone survey. The majority of the 244 respondents (n=202, 82.8%) initially chose to receive psychiatric care through video visits, while 13.5% (n=33) chose telephone visits and 1.2% (n=3) decided to postpone care until in-person visit availability. Patient age correlated with chosen visit type (*P*<.001; 95% CI 0.02-0.06). Patients aged ≥44 years were more likely than patients aged 0-44 years to opt for telephone visits (relative risk reduction [RRR] 1.2; 95% CI 1.06-1.35). Patient sex (*P*=.99), race (*P*=.06), type of insurance (*P*=.08), and number of previous visits to the clinic (*P*=.63) were not statistically relevant. Half of the respondents (132/244, 54.1%) stated theywere likely to continue with telepsychiatry even after in-person visits were made available. Telephone visit users were less likely than video visit users to anticipate future participation in telepsychiatry (RRR 1.08; 95% CI 0.97-1.2). Overall, virtual visits met or exceeded expectations for the majority of users.

**Conclusions:**

In this cohort, patient age correlates with the choice of virtual visit type, with older adults more likely to choose telephone visits over video visits. Understanding challenges to patient-facing technologies can help advance health equity and guide best practices for engaging patients and families through telehealth.

## Introduction

With the outbreak of COVID-19, many health care centers were forced to quickly modify their protocols and accept new modes of health care delivery. In-person routines and nonessential services were cancelled in an effort to protect both patients and frontline health care providers. New telehealth programs were swiftly implemented, and existing programs were expanded to provide ongoing access to care while simultaneously promoting physical distancing [1-3]. Federal and state governments as well as commercial insurers rapidly eased telehealth regulations [4]. The percentage of psychiatrists seeing more than three-quarters of their patient caseload via telehealth reached an unprecedented magnitude during the pandemic (85%) compared to before the pandemic (2.1%) [5]. This accelerated uptake contrasts starkly with the slow rate of engagement noted since the inception of telepsychiatry in 1956 [6-8].

For decades, telepsychiatry has enabled clinicians to deliver an array of mental health services in real time over virtual platforms. It provides health care access to a wider variety of populations residing in areas with limited community clinics, reduces the burden of disease, enhances models of integrated care, eliminates travel costs and logistics, and remains comparable in efficacy to conventional in-person mental health treatment [8,9]. Until recently, the uniform adoption of telepsychiatry by patients and families was limited by several barriers, including technology use, provider-patient relationships, and a lack of awareness of its existence [10].

Despite the rapid expanse of telepsychiatry during the COVID-19 pandemic, for most people, telepsychiatry was a novel and unfamiliar way of engaging in care [11]. Little is known about the factors influencing patients’ decisions to participate in telepsychiatry in the midst of a public health crisis. The aim of this paper is to examine the factors that influenced patients’ initial decisions to accept or decline telepsychiatry immediately after the issuance of the stay-at-home order in the US state of Michigan on March 23, 2020 [12], including choice of virtual care modality (video or telephone) and anticipated participation in telepsychiatry once clinics reopen for in-person visits. The findings will help provide actionable insights for further engagement of patients and families in telepsychiatry services.

## Methods

### Survey Setting and Participants

The Outpatient Psychiatry Clinics at the University of Michigan health care system, known as Michigan Medicine, provide approximately 67,000 psychiatry outpatient visits per year. In September 2019, the Outpatient Psychiatry Clinics launched a pilot telepsychiatry program with six providers; however, it failed to gain traction. By February 2020, only 26 virtual visits had been conducted via the Epic electronic medical record (EMR)–integrated platform. On March 23, 2020, in response to the COVID-19 pandemic, Governor Gretchen Whitmer issued a stay-at-home order (Executive Order 2020-21) [12] and Michigan Medicine called for the closure of all nonurgent ambulatory care clinics. Patients were offered a choice of video or telephone visits as well as the option to wait for an in-person visit when the clinics reopened. By June 2020, the Outpatient Psychiatry Clinics had converted 100% of in-person visits to virtual care, and the Psychiatry Department had become the lead department in telehealth for Michigan Medicine, followed by Neurology (62%) and Family Medicine (45%).

In April 2020, a multidisciplinary team from the Psychiatry Department and the Office of Patient Experience designed a telephone-based survey as part of a quality improvement (QI) project to evaluate the rapid scaleup of telepsychiatry and better understand patient experiences. The survey data were obtained as part of a strict QI initiative and as such did not require Institutional Review Board oversight. The telephone survey was conducted between June and August 2020 and targeted patients who had an in-person appointment date that fell in the first few weeks following the Michigan governor’s stay-at-home edict, necessitating conversion to virtual visits or deferment of in-person care. A total of 1030 patients who had an appointment scheduled between March 23 and April 13, 2020, were called, and 431 patients answered the call. Among these, 65 declined to participate in the survey, 113 asked to be recontacted at a more convenient time, and 9 were wrong numbers. A total of 244 patients participated in and completed the survey. This accounts for a response rate of 56.6% (231/244), which matches the overall response rates of Michigan Medicine (~50%) and the state (47.3%) for telephone surveys [13].

### Recruitment

The choice of telephone to contact participants offered a valuable opportunity to reach patients who lack the technological access required for a web-based or email survey. Seven volunteer interviewers, four medical students and three undergraduate students, completed a web-based Health Insurance Portability and Accountability Act (HIPAA) training module. They were instructed to follow Office of Patient Experience patient telephone call guidelines, which capped the maximum number of calls to each patient at two. They received a script to read at the beginning of each telephone call to give respondents the opportunity to make an informed decision and accept or decline the survey. Parents or proxies were interviewed on behalf of patients who were <11 years of age. In addition, adults were given the option to have a proxy answer the questionnaire on their behalf.

### Telephone-Based Questionnaire

The work group generated a 16-item questionnaire comprising 14 quantitative and 2 qualitative items, including the participant’s initial choice of visit type after the stay-at-home order, factors influencing this choice, digital platform use, experience with digital technology, future participation in telepsychiatry when clinics reopen for in-person visits, and readiness to resume in-person visits during the pandemic. We defined telepsychiatry as synchronous live mental health services delivered via video or telephone by a mental health clinician, such as a physician, nurse practitioner, psychologist, or social worker. An electronic version of the questionnaire was created using Qualtrics survey software. We adopted a quantitative approach by creating multiple-choice survey questions with predetermined answer options as well as Likert scale questions. Two open-ended questions were included to gather elaborated comments about respondents’ overall expectations of virtual visits and to welcome any additional reflections at the end of the survey. The participants’ demographics and number of previous clinic visits were extracted from the Epic EMR. The survey guide is included in Multimedia Appendix 1.

### Analysis

We used descriptive statistics to report individual survey responses and their relative percentages. We examined the association between chosen visit type and patient characteristics (age, sex, race, type of insurance, and number of previous visits to the clinic) and future virtual care participation using multivariable logistic regression through JMP 15 software (SAS Institute). Qualitative analysis of the free comments (questions 10 and 16) was not performed to allow for timely dissemination of our findings; however, narrative summaries of the comments are provided in the Results and Discussion sections.

## Results

### Survey Participants

Survey data were collected for a total of 244 patients whose original in-person appointments were scheduled in the first three weeks following the COVID-19 Michigan stay-at-home order. The majority of the 244 survey participants (n=212, 86.9%) were patients themselves; the remainder of respondents (n=32, 13.1%) were parents or proxies. In this sample of 244 respondents, 149 (61.1%) were adults aged 18-64 years, 49 (20.1%) were minors aged <18 years, and 45 (18.4%) were older people aged ≥65 years. Of the 244 respondents, 68.4% (n=167) identified themselves as female, 77.5% (n=189) as White, 10.7% (n=26) as Black, and 4.5% (n=11) as Asian. According to medical records, most survey respondents were established patients before the clinic closures who had attended 1-6 previous appointments (133/244, 54.5%) or >6 appointments (n=82, 33.6%) between July 1, 2019, and March 22, 2020. A total of 20/244 patients (8.2%) had no prior appointment with the clinic. Managed Medicaid and Medicare patients each represented 38 (15.6%) of the 244 visitors surveyed, 92 (37.7%) were insured with Blue Care Network (ie, Premier Care for Michigan Medicine employees), and 67 (27.5%) were insured with Blue Cross Blue Shield. In Michigan, straight Medicaid patients receive their behavioral health care through Community Mental Health clinics as part of their contract and are not routinely served by Michigan Medicine Outpatient Psychiatric Clinics. The patient characteristics are included in Multimedia Appendix 2.

### Telephone-Based Questionnaire

#### Participants’ Initial Choice of Visit Type After the Stay-at-Home Order 

Patients chose to receive psychiatric care through video visits with their provider (202/244, 82.8%), to receive psychiatric care through telephone visits (33/244, 13.5%), or to postpone care until in-person visit availability (3/244, 1.2%). Although some patients may have switched modalities for subsequent visits, survey respondents were asked to answer questions based on their experience with the initial modality they selected. Patient age correlated with initial choice in visit type (*P*=.002), and the magnitude of the association remained after adjustment for sex, race, type of insurance, and number of previous clinic visits (*P*<.001; 95% CI 0.02-0.06). Patients aged ≥44 years were more likely than patients aged 0-44 years to opt for telephone visits (relative risk ratio [RRR] 1.2; 95% CI 1.06-1.35). A slightly higher percentage of Asian respondents surveyed (10/11, 90.9%) chose to receive psychiatric care through video visits, followed by White respondents (159, 84.1%) and Black respondents (21/26, 80.7%); this difference was not statistically significant (*P*=.06). The patients’ sex (*P*=.99), race (*P*=.06), type of insurance (*P*=.08), and number of previous clinic visits (*P*=.63), delineating new and pre-existing patients, were not statistically relevant.

#### Factors Influencing Initial Choice of Video Visits

**Figure 1:**
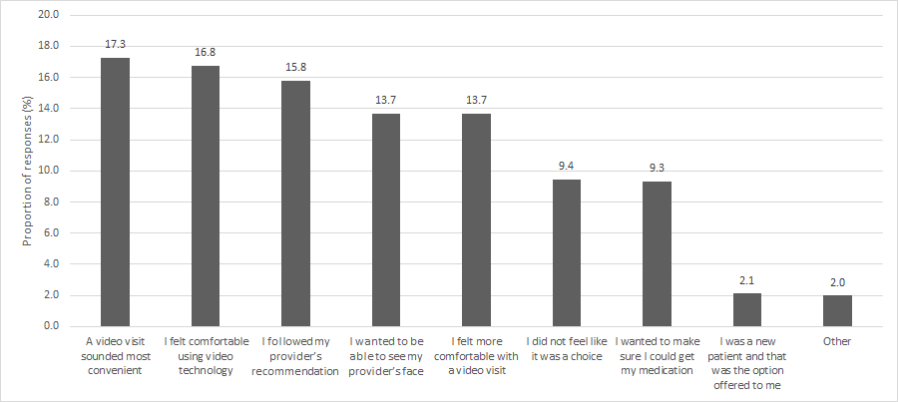
Factors influencing patients’ decisions to receive care by video visit.

Out of the 805 responses gathered, the main factors influencing the respondents’ decision to attempt a video visit included finding video visits more convenient (n=139, 17%), feeling comfortable with the technology (n=135, 17%), and feeling comfortable with a video visit in general (n=110, 14%). The influencing factors indicated by the respondents and their proportions are shown in Figure 1.

#### Factors Influencing Initial Choice of Telephone Visits

**Figure 2:**
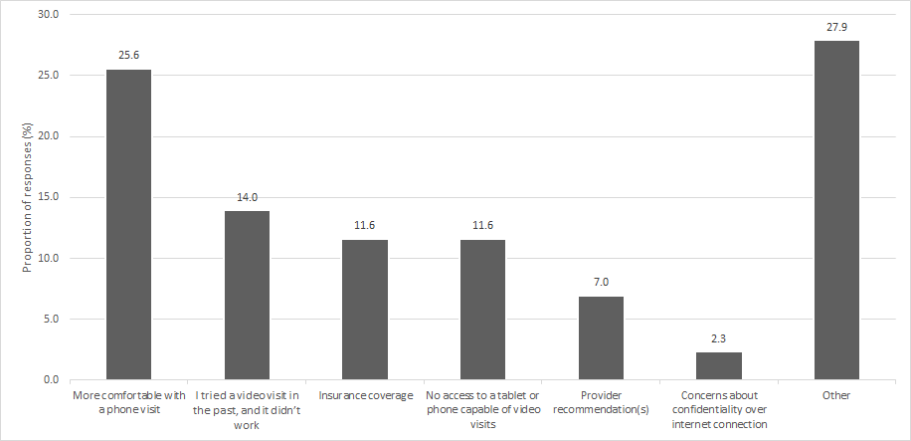
Factors influencing patients’ decisions to receive care by telephone visit.

A total of 43 responses were collected. Most respondents who chose telephone visits indicated that they felt more comfortable with the telephone (n=11, 26%), had complications with video visits in the past (n=6, 14%), or did not have appropriate technology for video (n=5, 12%) The influencing factors indicated by the respondents and their proportions are shown in Figure 2.

#### Factors Influencing Initial Choice of In-Person Visits

The 3 patients who opted to wait for in-person visits indicated a preference for face-to-face visits, with 1 participant (33%) also reporting lack of comfort with the video visit technology.

#### Participant Experiences With Virtual Visits

Among the 235 patients who elected to receive psychiatric care virtually (either video or telephone visits), 220 (93.6%) reported that virtual visits met or exceeded expectations (“as expected,” (n=126, 53.6%), “somewhat better than expected” (n=42, 17.9%), and “much better than expected” (n=52, 22.1%), with many participants indicating initial hesitation with virtual visits that were ultimately resolved with experience. 

#### Video Visits

Video visits were generally conducted via a Michigan Medicine Patient Portal application (149/202, 73.7%), although several other platforms were used. Use of the video technology was regarded by the 202 respondents as being either “extremely easy” (n=115, 56.9%), “somewhat easy” (n=41, 20.3%), “neither easy nor difficult” (n=16, 7.9%), “somewhat difficult” (n=23, 11.4%), or “extremely difficult” (n=7, 3.5%). Parents of pediatric patients commented that they preferred the video platform for virtual care because it was more engaging than telephone visits. 

#### Telephone Visits

Some patients commented that telephone visits were particularly advantageous when their internet connection was too unstable to access video technology or when the video platforms were otherwise problematic.

#### Anticipation of Virtual Care Participation After Clinics Open for In-Person Visits

**Figure 3:**
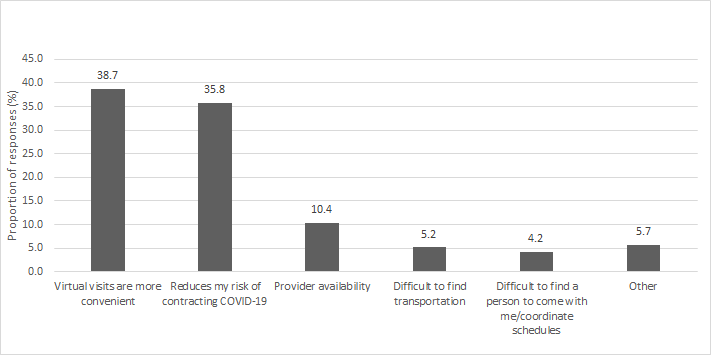
Factors influencing anticipated participation in virtual care after clinics open for in-person visits.

Approximately half of the respondents (132/244, 54.1%) stated that they were likely to continue with telepsychiatry visits even after in-person visits were made available. Patients who selected telephone visits were less likely than video visit users to anticipate participation in virtual care in the future (RRR 1.08; 95% CI 0.97-1.2). Factors influencing the decision to continue with virtual visits generated 235 answers and encompass convenience (n=149, 39%), reduced ability to contract COVID-19 (n=138, 36%) especially for those who reported having underlying medical conditions, and provider availability (n=40, 10%). Most patients who found the video technology extremely easy to use (115/202, 56.9%) indicated that they were either extremely likely (50/115, 43.5%) or somewhat likely (20/115, 17.4%) to continue with telepsychiatry visits after the clinics opened. Respondents who did not want to continue with virtual visits expressed a preference for face-to-face visits and a lack of comfort with the digital technology as the top two contributing factors. Parents of pediatric patients commented that their child had difficulty focusing and building a therapeutic relationship with their provider during virtual visits. The influencing factors indicated by the respondents and their proportions are shown in Figure 3.

#### Comfort With Returning to In-Person Visits During the COVID-19 Pandemic

All survey respondents were asked about their comfort with returning to in-person visits during the pandemic. Almost half of the patients (114/244, 46.7%) indicated that they were comfortable (ie, “extremely comfortable” or “somewhat comfortable”), while 105/244 (43.0%) were not. Among factors influencing patient comfort, perceived precautions by clinics to ensure COVID-19 protection accounted for 21% of the responses (182/885) followed by provider (140/885, 16%), state (105/885, 12%) or federal (71/885, 8%) recommendations regarding return to clinic, and provider availability (121/885, 14%). Several patients commented that the availability of a COVID-19 vaccine would influence their comfort with returning to in-person visits during the pandemic, while others wanted evidence of declining infection.

## Discussion

In this paper, we sought to examine factors influencing patients’ initial decisions to accept or decline telepsychiatry and choices of virtual care modality (telephone or video) after the Michigan COVID-19 stay-at-home order. Our findings showed that 235 of the 244 respondents (96.3%) rapidly decided to receive psychiatric care virtually, and among those, more than three-quarters (202/235, 86.0%) opted for video visits as their initial choice. Of the demographic factors explored, patient age correlated significantly with the initial choice of type of visit, while sex, race, insurance type, and number of previous visits did not.

A vast body of literature prior to the pandemic shows that virtual care cohorts to be largely younger adults, commonly below mid-forties [14-16], with a preference for video-based telehealth [16-18]. We observed a similar trend in our sample, with the majority of patients selecting video visits were aged 0-44 years and the majority of patients opting for telephone visits being aged ≥45 years. As previously published and as illustrated in our survey, key challenges to older patients’ willingness and readiness to partake in video visits encompass the capacity to use and access digital technology, low self-efficacy, and lack of support and facilitating conditions [17-19]. At Michigan Medicine, the GET Access (Geriatric Education for Telehealth Access) program serves as a model to address these challenges. Tailored virtual education successfully increased access to, comfort with, and participation in video visits for older adults.

One study showed that male patients were 1.6 times more likely than female patients with identical characteristics to use video than telephone calls for urgent care services [20]. Studies quantifying virtual care use and preference compared to traditional in-person care have yielded no consistent correlations with sex [14-16]. With the seriousness of the pandemic, preferences for a psychiatric visit type based on sex may have had less impact; this may also be true for race. Compared to other studies [16,21], our sample revealed no divide in selection of visit type with regard to patient race. This may stem from our cohort being predominantly White. Although health insurance type did not impact the initial choice of visit type, Medicare patients regardless of age are more likely to lack access to high-speed internet and smartphones with a data plan [22]. We advocate addressing these barriers with personalized support at different levels of use.

This survey was conducted at an unusual time, during a public health crisis, when many of the traditional barriers to scaling outpatient telehealth were significantly reduced. In a 2-year study conducted looking at primary care patients’ choices between video, telephone, and in-person visits before the pandemic (N=1.1 million), there were only 14% scheduled telehealth visits, with the majority (93%) tallied as telephone visits [16]. The pandemic may have served as a catalyst for more rapid acceptance of virtual care out of necessity. It has also opened doors for a blended care model bridging conventional psychiatric face-to-face sessions with telepsychiatry visits as deemed appropriate.

Our results yielded high anticipation of continuous participation in telepsychiatry even after in-person visits become available. Convenience was the most commonly cited reason to continue virtual care, exceeding but followed closely by reduced ability to contract COVID-19. This enthusiasm should compel state, federal, and private entities to strongly consider ongoing funding and regulatory models to support telepsychiatry both during the ongoing pandemic and beyond. In states such as Colorado and New Hampshire, lawmakers have already passed bills permanently supporting all or parts of the telehealth expansions they adopted during the COVID-19 pandemic [23]. To our surprise, anticipation of future participation in telepsychiatry was lower among patients who selected telephone visits compared to video visits, although the telephone seems to offer an easier communication medium for health care delivery. With three-quarters of our video visits taking place on the Epic EMR-integrated platform, use of video visits is contingent upon comfort not only with the video technology but also with the patient portal system as part of a broader telehealth platform.

Our initiative had several limitations. First, our sample lacked diversity with regard to race and insurance type, which may have concealed possible nuances in choice of visit type. Second, the respondents were not stratified by ZIP code. Individuals living in lower socioeconomic status neighborhoods or rural areas may favor telephone visits over video visits due to limited access to appropriate devices and high-speed internet. Finally, we surveyed patients three months after their appointment change. Their perceptions of virtual visits may have changed over time.

As we look toward the future and apply lessons learned regarding telepsychiatry, we recommend that payments and regulations match patient needs and preferences. Understanding challenges to patient-facing technologies, including patient attitudes and perceptions, will help advance health equity and guide best practices for engaging patients and families through telehealth. Further studies evaluating disadvantaged populations, such as straight Medicaid beneficiaries, would further inform health policy and should be designed to capture patients who do not have access to the technology required for conventional web-based or email surveys.

## Appendix

Multimedia Appendix 1 Patient survey guide regarding virtual visits.

Multimedia Appendix 2 Patient characteristics.

## Abbreviations

EMR: electronic medical record
GET Access: Geriatric Education for Telehealth Access
HIPAA: Health Insurance Portability and Accountability Act
QI: quality improvement
RRR: relative risk reduction
