# Correction: Factors Influencing Patients’ Initial Decisions Regarding Telepsychiatry Participation During the COVID-19 Pandemic: Telephone-Based Survey

**DOI:** 10.2196/27357

**Published:** 2021-01-27

**Authors:** Jennifer Severe, Ruiqi Tang, Faith Horbatch, Regina Onishchenko, Vidisha Naini, Mary Carol Blazek

**Affiliations:** 1 Department of Psychiatry University of Michigan Ann Arbor, MI United States; 2 University of Michigan Medical School Ann Arbor, MI United States

In “Factors Influencing Patients’ Initial Decisions Regarding Telepsychiatry Participation During the COVID-19 Pandemic: Telephone-Based Survey” (JMIR Formative Research 2020;4(12):e25469) the authors noted one error. In the originally published article, a value was incorrect in the Results section of the Abstract. The text read as follows:

Approximately half of the respondents (114/244, 46.7%) stated they were likely to continue with telepsychiatry even after in-person visits were made available.

This text has been revised to:

Half of the respondents (132/244, 54.1%) stated they were likely to continue with telepsychiatry even after in-person visits were made available.

The original Results section in its entierty is as follows:

A total of 244 patients whose original in-person appointments were scheduled within the first 3 weeks of the stay-at-home order in Michigan completed the telephone survey. The majority of the 244 respondents (n=202, 82.8%) initially chose to receive psychiatric care through video visits, while 13.5% (n=33) chose telephone visits and 1.2% (n=3) decided to postpone care until in-person visit availability. Patient age correlated with chosen visit type (P<.001; 95% CI 0.02-0.06). Patients aged ≥44 years were more likely than patients aged 0-44 years to opt for telephone visits (relative risk reduction [RRR] 1.2; 95% CI 1.06-1.35). Patient sex (P=.99), race (P=.06), type of insurance (P=.08), and number of previous visits to the clinic (P=.63) were not statistically relevant. Approximately half of the respondents (114/244, 46.7%) stated they were likely to continue with telepsychiatry even after in-person visits were made available. Telephone visit users were less likely than video visit users to anticipate future participation in telepsychiatry (RRR 1.08; 95% CI 0.97-1.2). Overall, virtual visits met or exceeded expectations for the majority of users.

The revised Results section of the abstract in its entirety is as follows:

A total of 244 patients whose original in-person appointments were scheduled within the first 3 weeks of the stay-at-home order in Michigan completed the telephone survey. The majority of the 244 respondents (n=202, 82.8%) initially chose to receive psychiatric care through video visits, while 13.5% (n=33) chose telephone visits and 1.2% (n=3) decided to postpone care until in-person visit availability. Patient age correlated with chosen visit type (P<.001; 95% CI 0.02-0.06). Patients aged ≥44 years were more likely than patients aged 0-44 years to opt for telephone visits (relative risk reduction [RRR] 1.2; 95% CI 1.06-1.35). Patient sex (P=.99), race (P=.06), type of insurance (P=.08), and number of previous visits to the clinic (P=.63) were not statistically relevant. Half of the respondents (132/244, 54.1%) stated they were likely to continue with telepsychiatry even after in-person visits were made available. Telephone visit users were less likely than video visit users to anticipate future participation in telepsychiatry (RRR 1.08; 95% CI 0.97-1.2). Overall, virtual visits met or exceeded expectations for the majority of users.

The correction will appear in the online version of the paper on the JMIR Publications website on January 27, 2021, together with the publication of this correction notice. Because this was made after submission to PubMed, PubMed Central, and other full-text repositories, the corrected article has also been resubmitted to those repositories

