# Supplementary material for: Factors Influencing Patients’ Initial Decisions Regarding Telepsychiatry Participation During the COVID-19 Pandemic: Telephone-Based Survey
Source: JMIR Form Res. 2020 Dec 22;4(12):e25469. doi: 10.2196/25469 (PMC7758083; doi:10.2196/25469)
Supplement: Multimedia Appendix 1 [file formative_v4i12e25469_app1.docx]

# Patient Survey Guide Regarding Virtual Visits

# Purpose:

1. Understand why patients either accepted or declined a virtual visit when they were asked to convert in-person appointments due to the COVID-19 pandemic.
2. Identify the patient’s interest and ease in adapting to virtual visits.
3. Assess willingness to come back to face-to-face visits.

# Survey population:

- Patients who had in-person appointments that were scheduled to occur in March and April who were contacted and asked to move the visit to a phone or video visit.
- This includes new patient video-only visits as well as new patients who were on the waiting list and offered a new patient visit via video;
  - Also includes patients who declined because they couldn’t/didn’t want to receive care through video visits.

# Guidelines:

Please conduct patient phone calls between 10:00am and 6:00pm

If you are unable to connect with the patient, leave a message describing the purpose of call and state that you will try calling back. Document the time you called. After completing the rest of the list, try again during a different time of day.

Please do not contact a patient more than twice.

# Prior to calling the patient:

Q: Does the patient utilize an interpreter?

- 1. Yes
  2. No

[If Yes, utilize the M-TERP line for interpretive assistance (734-936-8377)]

# Patient Survey Script & Questions:

***[INTERVIEWER]:*** *Hello, my name is XXXXXX. May I speak to [name of participant] please?*

***(After confirmation of correct participant):*** *I am a medical student from the University of Michigan working with the Outpatient Psychiatry Clinic. We are conducting a brief survey of our patients about their experience with transitioning from in-person appointments to virtual visits during the Stay-at-Home orders. The survey will help us improve the virtual visit experience and help us create a smooth transition back to in-person care. Participation in the survey is confidential and all responses will remain anonymous. The survey takes less than 10 minutes to complete, would you be interested in participating?*

**If no:** *Thank you for your time. Have a great day [hang up and note that patient declined]*

**If yes:** *Thank you for your willingness to participate. As a reminder, the survey is confidential and anonymous. If you do not wish to answer a question at any time you are welcome to skip to the next question. Also, if you wish to stop the survey simply let me know and we can conclude at your request.*

***[INTERVIEWER]:*** *Most questions are multiple choice. I will read you the question and give you the response options. Please let me know if you need any question repeated or if you have any clarifying questions. Are you ready to begin?*

## Questions:

1. Who is responding to the survey?
   1. Patient
   2. Parent/proxy
2. When you were contacted about changing your appointment to a virtual visit due to the COVID-19 pandemic, what did you decide to do?
   1. Receive care through a video visit
   2. Receive care through a telephone visit
   3. Wait for an in-person visit
   4. Other: _________________
3. [If 2a] Which of the following factors contributed to your decision to choose a video visit? (select all that apply)
   1. I did not feel that it was a choice
   2. I was a new patient and that was the option offered to me
   3. I wanted to make sure I could get my medication
   4. I wanted to be able to see my provider’s face
   5. I felt more comfortable with a video visit
   6. I felt comfortable using video technology
   7. I followed my provider’s recommendation
   8. A video visit sounded most convenient
   9. Other: __________________
4. [If 2a] What platform did you use for your video visit?
   1. Patient portal
   2. Zoom
   3. Blue Jeans
   4. Doximity
   5. Other: ____________________
   6. I don’t remember
5. [If 2a] How difficult was it for you to use the technology required for the video visit?
   1. Extremely easy
   2. Somewhat easy
   3. Neither easy nor difficult
   4. Somewhat difficult
   5. Extremely difficult
6. [If 2a] How did the virtual visit compare to your expectations?
   1. Much better than expected
   2. Somewhat better than expected
   3. As expected
   4. Somewhat worse than expected
   5. Much worse than expected
7. [If 2a] Do you have any comments you’d like to share about your expectations?
8. [If 2a] Once the clinic opens for in-person visits, how likely are you to continue virtual visits?
   1. Extremely likely
   2. Somewhat likely
   3. Neither likely nor unlikely
   4. Somewhat unlikely
   5. Extremely unlikely

[Skip to Q14]

1. [If 2b] Which of the following factors contributed to your decision to choose a telephone visit instead of a video visit? (select all that apply)
   1. I tried a video visit in the past, and it didn’t work
   2. Insurance coverage
   3. No access to a tablet or phone capable of video visits
   4. Concerns about confidentiality over internet connection
   5. More comfortable with a phone visit
   6. Provider recommendation(s)
   7. Hearing and/or vision impairment
   8. Other: __________________
2. [If 2b] How did the virtual visit compare to your expectations?
   1. Much better than expected
   2. Somewhat better than expected
   3. As expected
   4. Somewhat worse than expected
   5. Much worse than expected
3. [If 2a] Do you have any comments you’d like to share about your expectations?
4. [If 2a] Once the clinic opens for in-person visits, how likely are you to continue virtual visits?
   1. Extremely likely
   2. Somewhat likely
   3. Neither likely nor unlikely
   4. Somewhat unlikely
   5. Extremely unlikely

[Skip to Q14]

1. [If 2c] Which of the following factors contributed to your decision to wait for an in-person appointment? (select all that apply)
   1. No insurance
   2. No insurance coverage for video or phone visit
   3. No internet access
   4. No access to a tablet or phone capable of video visits
   5. Lack of comfort with the technology or video visit
   6. Concerns about confidentiality over phone or internet connection
   7. No private space to talk
   8. Prefer face-to-face visits
   9. Hearing and/or vision impairment
   10. Other: ___________________

[Skip to Q15]

1. [If 2a or b] What are some reasons why you **would** want to continue virtual visits instead of in-person visits? (select all that apply)
   1. Virtual visits are more convenient (save time, save money, suitable for my health/mental health condition, etc.)
   2. Reduces my risk of contracting COVID-19
   3. Difficult to find transportation
   4. Difficult to find childcare
   5. Difficult to find a person to come with me/coordinate schedules
   6. Provider availability
   7. Other: ___________________

[Skip to Q15]

1. [If 2a, b, or c] During the ongoing COVID-19 pandemic, how comfortable are you with returning to in-person visits in the clinic?
   1. Extremely comfortable
   2. Somewhat comfortable
   3. Neither comfortable nor uncomfortable
   4. Somewhat uncomfortable
   5. Extremely uncomfortable
2. If 2a, b, or c] During the ongoing COVID-19 pandemic, what factors would influence your comfort with returning to in-person visits? (check all that apply)
   1. Insurance coverage
   2. State government direction/orders
   3. National government direction/orders
   4. Provider recommendation(s)
   5. Provider availability
   6. Precautions taken to protect patients from COVID-19
   7. Transportation availability
   8. Childcare availability
   9. Work or personal schedule
   10. Other: ___________________
3. Please share any additional comments you may have about virtual visits.

***[INTERVIEWER]:*** *This completes the survey, thank you again for your participation. Your responses will help our department improve virtual care for all patients.*

*Have a good day.*

1. Interviewer Name: ____________________

# Additional elements to enter from the patient’s record:

1. When the patient was contacted about converting the appointment to a virtual visit, was the patient a new or pre-existing patient with the clinic?
   1. New patient
   2. Pre-existing patient
2. Scheduling Department
   1. EAA GERIATRIC PSYCH
   2. RUB PSYCH ADULT
   3. RUB PSYCH ATS
   4. RUB PSYCH BEHAV SLEEP
   5. RUB PSYCH CHILD ADOL
   6. RUB PSYCH GERIATRICS
3. Provider type
   1. Limited License Social Worker
   2. Master's Limited Licensed Psychologist
   3. Nurse Practitioner
   4. Physician
   5. Physician Assistant
   6. Psychologist
   7. Psychology Resident (post-doc)
   8. Resident
   9. Resource
   10. Social Work Fellow
   11. Social Worker
   12. Social Worker Student
4. Provider name (this is the provider for whom the appointment was originally scheduled with when the patient was contacted): ______________________
5. Age
   1. Under 12
   2. 12 - 17
   3. 18 - 24
   4. 25 - 34
   5. 35 - 44
   6. 45 - 54
   7. 55 - 64
   8. 65 - 74
   9. 75 - 84
   10. 85 or older
6. Sex
   1. Male
   2. Female
7. Ethnicity
   1. Hispanic
   2. Non-Hispanic
   3. Patient Refused
   4. Unknown
8. Race
   1. White or Caucasian
   2. Black or African American
   3. American Indian or Alaska Native
   4. Asian
   5. Native Hawaiian or Pacific Islander
   6. Other
   7. Unknown
   8. Patient Refused
9. Zip Code: _______________
10. Primary Insurance
    1. AAA
    2. Aetna
    3. Aetna Better Health
    4. Allied Benefit Systems
    5. Allstate
    6. ASR Corp
    7. Auto Insurance Other
    8. Auto Owners
    9. Blue Cross Blue Shield (BCBS)
    10. Blue Care Network/UM Premiere Care (BCN)
    11. Beacon Health Options
    12. Benesys
    13. Buckeye Health Plan
    14. Champva
    15. Cigna
    16. Citizens
    17. Cofinity Other
    18. Commercial Generic
    19. Dept of Veterans Affairs
    20. Employee Benefits Logistics
    21. Farmers Insurance
    22. Frontpath Health Other
    23. Golden Rule
    24. HAP
    25. Humana
    26. Huron Valley Pace
    27. JFP Benefit
    28. Liberty Union
    29. Lifetrac
    30. McLaren
    31. Medicare
    32. Meridian Health Plan
    33. Meritain Health
    34. Michigan Complete Health
    35. Molina Healthcare
    36. Nippon Life
    37. Paramount
    38. Physicians Health Plan of Mid-Michigan (PHPMM)
    39. Priority Health
    40. Skilled Nursing Facility
    41. State Farm
    42. Tricare East
    43. Trustmark Coresource
    44. UMR
    45. UMR Payor ID 39026
    46. UnitedHealthcare
    47. UnitedHealthcare Community Pln
    48. UnitedHealthcare Student Resources
    49. Upper Peninsula Health Plan
    50. US Health and Life
    51. Varipro
    52. Wellcare
    53. Workers Com Other
    54. Other: ________________
