# Supplementary material for: Factors Influencing Patients’ Initial Decisions Regarding Telepsychiatry Participation During the COVID-19 Pandemic: Telephone-Based Survey
Source: JMIR Form Res. 2020 Dec 22;4(12):e25469. doi: 10.2196/25469 (PMC7758083; doi:10.2196/25469)
Supplement: Multimedia Appendix 2 [file formative_v4i12e25469_app2.docx]

**Table 1. Patient Characteristics (N=244)**

| **Characteristics** | **Participants** |
| --- | --- |
| **Age (years), n (%)** | |
| <12 | 17 (7) |
| 12-17 | 32 (13) |
| 18-24 | 27 (11) |
| 25-34 | 38 (16) |
| 35-44 | 35 (14) |
| 45-54 | 27 (11) |
| 55-64 | 22 (9) |
| 65-74 | 36 (15) |
| 75-84 | 8 (3) |
| ≥85 | 1 (<1) |
| No response |  |
| **Gender, n (%)** | |
| Female | 167 (68) |
| Male | 72 (30) |
| No response | 5 (2) |
| **Race, n (%)** | |
| White or Caucasian | 189 (77) |
| Black or African American | 26 (11) |
| Asian | 11 (5) |
| Native Hawaiian or Pacific Islander | 2 (1) |
| American Indian or Alaska Native | 1 (<1) |
| Other | 11 (5) |
| Patient Refused | 3 (<1) |
| No response | 1 (<1) |
| **Ethnicity, n (%)** | |
| Non-Hispanic | 225 (92) |
| Hispanic | 10 (4) |
| Unknown | 5 (2) |
| Patient Refused | 3 (1) |
| No response | 1 (<1) |
| **Patient Status, n (%)** | |
| New patient | 8 (3) |
| Pre-existing patient | 235 (96) |
| No response | 1 (<1) |
